# Weight change across adulthood in relation to the risk of COPD

**DOI:** 10.1265/ehpm.25-00059

**Published:** 2025-08-08

**Authors:** Entong Gong, Ziwei Kou, Yinan Li, Qinghai Li, Xinjuan Yu, Tao Wang, Wei Han

**Affiliations:** 1 Graduate School, Dalian Medical University, Dalian, China; 2 Department of Respiratory and Critical Care Medicine, Qingdao Hospital, University of Health and Rehabilitation Sciences (Qingdao Municipal Hospital), Qingdao, China; 3 Department of Respiratory and Critical Medicine, Affiliated Hospital of Shandong Second Medical University, Weifang, China; 4 Department of Medicine, Qingdao University, Qingdao, China; 5 School of Health and Life Sciences, University of Health and Rehabilitation Sciences, Qingdao, China; 6 Department of Respiratory and Critical Medicine, Department of Emergency, Department of General Practice, Qingdao Key Laboratory of Common Diseases, Qingdao Hospital, University of Health and Rehabilitation Sciences (Qingdao Municipal Hospital), Qingdao, China

**Keywords:** Weight change, Obesity, NHANES, Chronic obstructive respiratory disease

## Abstract

**Background:**

Despite some studies identifying a potential association between obesity and chronic obstructive pulmonary disease (COPD) risk, previous research had overlooked the dynamic nature of body weight over time, leading to inconsistent findings. The purpose of this study is to elucidate the relationship between adult weight change and COPD risk by adjusting for potential confounding factors.

**Methods:**

We conducted a retrospective analysis using data from ten NHANES cycles (1999–2018), including adults aged 40–74 years. Weight change patterns were assessed using BMI at three time points and classified into five categories per period. Absolute weight change was also grouped into five levels. Multivariate logistic regression models, incorporating sampling weights, were used to examine associations between weight change and COPD, adjusting for demographic and lifestyle covariates.

**Results:**

Compared with participants who maintained normal weight, stable obesity participants had increased risk of COPD from age 25 years to 10 years before the survey (OR = 1.45, 95% CI = 1.15 to 1.83), in the 10 years period before the survey (OR = 1.75, 95% CI = 1.47 to 2.08), and from age 25 years to survey (OR = 1.84, 95% CI = 1.46 to 2.31). Three periods indicate that weight gain in adulthood was associated with risk of COPD. In addition, substantial weight gain of more than 20 kg was associated with a higher risk of COPD. In stratified analyses, we also observed a more significant association between weight change and the risk of COPD in never smokers compared to former smokers.

**Conclusions:**

Our study suggested that stable obesity and weight gain in adulthood were associated with an increased risk of COPD compared to those who maintain a normal weight, and that the association between weight gain and the incidence of COPD appears closer in patients who have never smoked.

**Supplementary information:**

The online version contains supplementary material available at https://doi.org/10.1265/ehpm.25-00059.

## Graphical Abstract

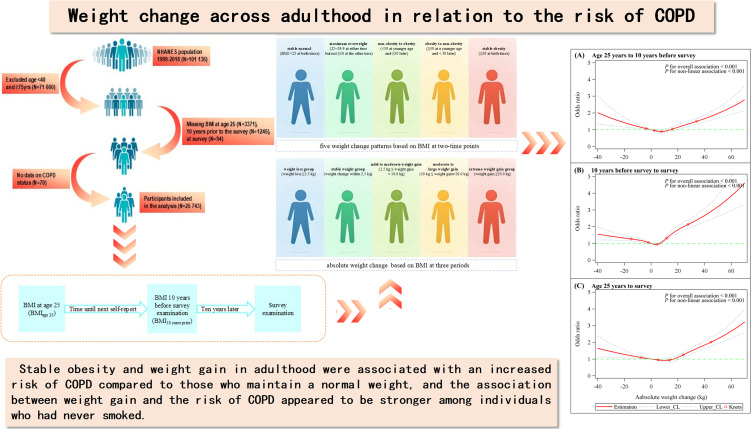

## Introduction

Chronic obstructive pulmonary disease (COPD) is a common chronic respiratory disease (CRD) worldwide. According to relevant reports, 3.2 million people worldwide died from COPD in 2015, an increase of 11.6% compared to 1990 [1]. In addition, there were 212.3 million cases of COPD in 2019, which was the main cause of death in CRDs, accounting for 3.3 million deaths [2]. Due to the high morbidity rate and long duration of COPD, it has increasingly recognized as an important contributor to the global burden of non-communicable diseases and as one of the leading causes of morbidity and mortality worldwide. Therefore, it is particularly important to find out the related causes of COPD and prevent the occurrence of the disease in advance.

Existing evidence suggested that obesity was associated with poorer lung function in COPD patients, and obese individuals might have a higher risk of COPD [3]. A cohort study on elderly COPD patients in Australia showed that 42% of participants were obese and 35% were overweight [4]. In addition, a multi center prospective cohort study of Genetic Epidemiology of COPD had clearly demonstrated that obesity was associated with increased morbidity in moderate to severe COPD [5]. Many previous studies had shown that high BMI was strongly associated with COPD.

On the other hand, malnutrition and low BMI among patients diagnosed with COPD was also thought to be important contributing factors [6–8]. Evidence from a survey on the nutritional status of patients with COPD found that underweight and weight loss contribute to the risk of exacerbation and progress stage of COPD [9]. It had also been reported that low BMI was a strong predictor of mortality in patients with COPD [10], and in several retrospective survival studies, it was found that weight loss [11] and low body weight [12, 13] were inversely correlated with survival rate. In addition, there were differences from the above research results, Jos Slangen et al. through two survival analyses found that higher and lower body weight were associated with better survival rates in COPD patients [14]. These research results were inconsistent, possibly due to differences in gender, age, lifestyle and socio-economic status of the study population, but all suggested a correlation between obesity or weight loss and the risk of COPD. However, previous studies had mostly used a single BMI and largely ignored the dynamics of weight over time. Therefore, more researches were needed to evaluate the long-term impact of weight changes on COPD in certain life cycles.

Weight change is an important factor affecting physical and mental health, including weight gain and loss. For most people, excessive obesity often accumulates in the early and middle stages of adulthood. The National Health and Nutrition Examination Survey (NHANES) regularly collects information on participants’ weight history and COPD diagnosis. NHANES is a national health database based on the US population, which is implemented by the National Center for Health Statistics (NCHS) under the US Centers for Disease Control and Prevention (CDC). Using these data, we aim to investigate the association between weight change and COPD from young adulthood to middle age and late adulthood.

## Methods

### Study design and population

NHANES is a national health monitoring plan led by the NCHS. Through a complex stratified, multi-stage probability sampling design, NHANES selected a representative sample of the non institutional American population to assess the health and nutrition status of the non institutional residents in the United States. Since 1999, NHANES has been conducting annual continuous data collection through methods such as household questionnaires, mobile medical center (MEC) physical examinations, and laboratory testing. This project adopts a multi-stage probability sampling design, using nationally representative samples to oversample certain key populations such as ethnic minorities, the elderly, and low-income groups to improve statistical accuracy. NHANES provides critical data support for public health policy-making and health research. The survey was approved by the Research Ethics Review Committee of NCHS and written informed consent was provided to all participants during the survey period [15].

This study used data from ten consecutive cycles (1999–2000 to 2017–2018) of NHANES, including adults aged 40–74 in the NHANES survey [16]. We excluded participants under 40 and over 74 years old, those who did not have complete COPD status data, and those who did not have height or weight information at age 25 years and/or 10 years before the survey and/or at the time of the survey. The purpose is to ensure that the research population is more homogeneous. Previous research suggests that weight change patterns and COPD risks vary significantly across different life stages, and excluding individuals outside this age range allowed for more consistent and interpretable results. We created a retrospective study based on cross-sectional data using recall questions about weight history and age during COPD diagnosis. Specifically, self-reported weight changes were evaluated based on weight recall of participants at age 25 years and 10 years before the survey.

Due to the limitations of available data in the NHANES dataset, COPD was defined based on self-reported physician diagnoses of emphysema, chronic bronchitis and COPD, rather than objective lung function tests. If the participant answers “yes” to the following question in the standardized medical condition questionnaire conducted during personal interviews: “Have you been informed that you have emphysema/chronic bronchitis/COPD?”, it was recorded as having COPD. The research design is visually described in Fig. S1. It is important to note that while this definition aligns with the available data in NHANES, it may not be as precise as clinical definitions based on spirometry, potentially leading to a misclassification bias. Finally, we retained 25743 samples in our queue for analysis.

### Assessments of weight change

The subjects measured their height and weight during the physical examination and were asked to recall their weight at age 25 years and 10 years before the survey. The BMI at these three time points was calculated as corresponding weight (kg) divided by the square of the survey height (m^2^). BMI was further classified as underweight (<18.5 kg/m^2^), normal weight (18.5–24.9 kg/m^2^), overweight (25.0–29.9 kg/m^2^) and obese (≥30.0 kg/m^2^). This is the weight classification standard based on BMI proposed by the World Health Organization (WHO). NHANES has routinely asked questions about weight histories, including weight at age 25 and weight 10 years before the survey, alongside weight at the survey [16]. Based on previous research, we have created three periods of weight change patterns: BMI at age 25 to 10 years before the survey, BMI at age 25 to the survey and BMI at 10 years before the survey to BMI at the survey. Using BMI at two-time points, we defined five weight change patterns for each period: stable normal (BMI <25 at both times), maximum overweight (25–29.9 at either time but not ≥30 at the other time), non-obesity to obesity (<30 at younger age and ≥30 later), obesity to non-obesity (≥30 at a younger age and <30 later) and stable obesity (≥30 at both times). The method has been described in detail in a previous publication using NHANES data [16, 17]. To compare with other studies [17, 18], absolute weight change at three periods was also divided into five groups: weight loss group (weight loss ≥2.5 kg), stable weight group (weight change within 2.5 kg), mild to moderate weight gain (2.5 kg ≤ weight gain < 10.0 kg), moderate to large weight gain (10 kg ≤ weight gain < 20.0 kg), and extreme weight gain group (weight gain ≥20.0 kg). This grouping method has been reasonably applied in multiple articles [19, 20].

### Covariates

Potential confounding factors, including gender, survey age, race/ethnicity, family income-to poverty ratio (PIR), education level, smoking and drinking status were obtained through demographic questionnaires. Race/ethnicity is divided into Mexican American, non-Hispanic white, non-Hispanic black, other Hispanic, and other. Education level is divided into less than high school, high school or equivalent, college or above. Family PIR is calculated based on poverty guidelines by dividing household income and further dividing it into four categories (0–1.3, –1.8, –3.0 and >3.0). Lifestyle factors include alcohol consumption (non-drinker, low to moderate drinker, and heavy drinker) and smoking status (never, former, current smoker). A drinker was defined as any participant who consumed at least 12 alcoholic beverages of any type in any given year [21].

### Statistical analysis

All analyses incorporated sampling weights, stratification, and primary sampling units (PSUs) to explain NHANES’s complex sampling design and provide nationally representative estimates. For continuous variables, data for population characteristics are represented by mean and standard deviation (SD), while the categorical variables are presented as frequency (n) and proportion (%).

Using a multivariate logistic regression model to explore the independent relationship between weight status or weight change patterns and COPD. All other patterns of weight change were compared to the stable normal pattern. The first model (Model 1) did not adjust for potential confounding factors. We adjusted the survey age and gender in Model 2. In Model 3, we further adjusted for race/ethnicity, education level, family PIR, smoking status, and alcohol consumption. Use dummy variables to represent missing data for covariates. Perform predefined subgroup analysis and potential modification effects based on survey age (<50 years and ≥50 years), gender (male and female), smoking status (never smoking and ever smoking), and baseline BMI (underweight/normal weight and overweight/obesity).

We also investigated the relationship between absolute weight change group and the risk of COPD. Absolute weight changes were also considered as continuous variables to test the robustness of our results. All other patterns of weight change were compared with the stable weight group.

We also used restricted cubic spline with 5 nodes at points 5, 25, 50, 75, and 95 to evaluate the dose-response relationship. The adjusted covariates in the restricted cubic spline were the same as the covariates adjusted in Model 3 of the logistic regression.

All statistical analyses were conducted using SAS 9.4 (SAS Institute Inc., Cary, NC, United States) in 2024. The forest map was created by the “forestplot” package in R 4.0.2 (R Foundation for Statistical Computing, Vienna, Austria). Statistical significance was defined as *p* < 0.05 using two-sided tests.

## Results

### Baseline characteristics and weight change pattern

Table 1 presents the characteristics of weight change patterns among study participants in NHANES at age 25 to 10 years before the survey. The proportion of participants in the study divided into five weight change patterns during this period: the stable normal group had the highest proportion with 37.7% of participants, the maximum overweight group accounting for 35.7%, the stable obesity group accounting for 6.3% and 19.3% of participants transitioning from non-obesity to obesity in adulthood, with an average weight gain of 24.8 kg. Only 0.9% of participants transitioned from obesity to non-obesity in adulthood, with an average weight loss of 17.1 kg. The average age of the study population was 56 years old, and 51.5% were female. There were 5.7% Mexican American, 72.8% non-Hispanic white, 10.5% non-Hispanic black, 4.5% other Hispanic and 6.5% other people in our study.

**Table 1:** According to weight change patterns from age 25 years to 10 years before survey.^a^

**Characteristics**	**Total**	**Stable Normal**	**Maximum Overweight**	**Non-obesity to Obesity**	**Obesity to ** **Non-obesity**	**Stable Obesity**	***P* value**
**Participants**	25743	9022 (37.69)	9483 (35.74)	5300 (19.33)	268 (0.90)	1670 (6.34)	
**Age (mean ± SD^a^, years)**	55.92 ± 9.90	54.04 ± 9.77	56.73 ± 9.89	58.74 ± 9.39	54.68 ± 9.72	52.73 ± 9.32	<0.001
**Female**	12980 (51.54)	5434 (64.60)	3954 (40.67)	2660 (48.43)	116 (45.10)	816 (45.58)	<0.001
**Race/ethnicity**							
Mexican American	4163 (5.73)	1116 (4.32)	1731 (6.46)	987 (6.62)	64 (8.84)	265 (6.82)	<0.001
Other Hispanic	2140 (4.52)	682 (4.35)	890 (5.13)	434 (3.98)	24 (5.84)	110 (3.56)	
Non-Hispanic White	11214 (72.79)	4124 (73.35)	4014 (72.29)	2298 (73.88)	112 (70.43)	666 (69.28)	
Non-Hispanic Black	5849 (10.49)	1830 (9.22)	2071 (10.19)	1341 (11.53)	59 (12.69)	548 (16.32)	
Other	2377 (6.47)	1270 (8.76)	777 (5.93)	240 (3.99)	9 (2.20)	81 (4.02)	
**Education**							
Less than high school	6637 (15.52)	2105 (14.53)	2553 (16.27)	1445 (15.79)	111 (24.78)	423 (15.10)	<0.001
High school or equivalent	5980 (24.05)	2033 (22.47)	2199 (24.67)	1274 (25.22)	44 (18.71)	430 (27.15)	
College or above	13111 (60.42)	4876 (63.00)	4726 (59.06)	2580 (58.99)	113 (56.51)	816 (57.76)	
**Family income-poverty ratio level**							
0∼1.3	6240 (16.09)	2120 (15.83)	2198 (14.99)	1338 (16.68)	108 (30.50)	476 (19.92)	<0.001
∼1.85	2877 (8.68)	949 (8.05)	1062 (8.33)	628 (9.61)	30 (10.28)	208 (11.41)	
∼3	4173 (16.58)	1375 (15.38)	1530 (16.82)	946 (18.34)	35 (13.83)	287 (17.33)	
>3	10166 (58.65)	3739 (60.73)	3866 (59.86)	1927 (55.37)	81 (45.39)	553 (51.35)	
**Smoking status**							
Never smoker	12819 (50.02)	4457 (49.63)	4706 (49.88)	2685 (50.71)	100 (37.13)	871 (52.77)	<0.001
Former smoker	7395 (29.56)	2246 (26.13)	2882 (31.42)	1741 (33.08)	86 (31.07)	440 (28.51)	
Current smoker	5518 (20.42)	2314 (24.24)	1891 (18.70)	873 (16.20)	82 (31.80)	358 (18.72)	
**Drinking status**							
Non-drinker	6904 (24.60)	2191 (21.97)	2450 (24.05)	1664 (29.06)	76 (27.47)	523 (29.50)	<0.001
Low to moderate drinker	8541 (42.21)	3046 (42.54)	3230 (42.98)	1725 (42.65)	70 (33.33)	470 (35.80)	
Heavy drinker	7073 (33.18)	2626 (35.50)	2653 (32.96)	1233 (28.29)	89 (39.20)	472 (34.70)	
**Body mass index (mean ± SD^a^)**							
At age 25 years	23.61 ± 4.66	20.67 ± 2.06	23.48 ± 2.73	24.71 ± 3.11	34.31 ± 6.15	35.02 ± 5.75	<0.001
At 10 years before survey	27.71 ± 6.28	22.27 ± 1.97	27.13 ± 1.62	34.39 ± 4.71	26.84 ± 2.55	39.33 ± 7.88	<0.001
**Absolute weight change (mean ± SD^a^, kg)**	10.31 ± 13.09	3.78 ± 5.30	9.08 ± 8.18	24.81 ± 14.73	−17.12 ± 16.41	11.06 ± 18.78	<0.001

^a^Categorical variables were presented as weighted numbers (weighted %), while continuous variables were described as mean ± SD (standard deviation).

The average BMI of the study participants at the age of 25 was 23.6 kg/m^2^, while the average BMI for the 10 years before the survey was 27.7 kg/m^2^, and at the time of the survey was 29.6 kg/m^2^ (Table 1; Table S1). According to the average level, participants gained 10.3 kg in absolute weight from the age of 25 to the 10 years before the survey, 5.1 kg in absolute weight during the 10 years before the survey, and 15.4 kg in weight from the age of 25 to the survey (Table 1; Table S1, S2). The self-report of the study population showed that during the 10 years before the survey, 17.5% of participants transitioned from non-obesity to obesity, and 4.3% of participants lost weight from obesity to non-obesity (Table S1). From the age of 25 to the survey, the proportion of participants who gained weight increased to 32.7%, while the proportion of participants who lost weight decreased to 1.1% (Table S2).

### Associations of weight status with COPD

Among 25743 participants, 2348 were diagnosed with COPD, accounting for 9.1%. By observing and evaluating the BMI status at three time points, we found a significant correlation between obesity and COPD risk. Obesity patients had an OR of 1.40 (95% CI = 1.14 to 1.73) at the age of 25, while the ORs for the 10 years before the survey and at the time of the survey were 1.50 (95% CI = 1.27 to 1.76) and 1.83 (95% CI = 1.58 to 2.12), respectively (Table S3). In addition, by examining the dose-response relationship between BMI and the risk of COPD, we found a significant U-shaped correlation (*p* nonlinearity <0.001) (Fig. S2).

### Associations of weight change patterns with COPD

Table 2 reported the relationship between adult weight change patterns and COPD at three time periods, with the stable normal group as the control group. From the age of 25 to 10 years before the survey, study participants with stable obesity in adulthood were associated with 45% increase in the risk of COPD (OR = 1.45, 95% CI = 1.15 to 1.83). The OR for 10 years before the survey was 1.75 (95% CI = 1.47 to 2.08), and the OR for the age of 25 to the survey was 1.84 (95% CI = 1.46 to 2.31). Participants who increased their weight from non-obesity to obesity also showed a significant correlation with the risk of COPD, with an OR of 1.49 (95% CI = 1.27 to 1.75) from age 25 years to 10 years before the survey, 1.76 (95% CI = 1.47 to 2.11) in the 10 years period before the survey, and 1.73 (95% CI = 1.48 to 2.02) from age 25 years to the survey.

**Table 2:** Odd ratios (OR) and 95% confidence intervals (CIs) of COPD with weight change patterns.^a^

**Weight Change Patterns^b^**	**No. of COPD/No. ** **of participants**	**Model 1^c^**		**Model 2^d^**		**Model 3^e^**	

		**OR (95% CI)**	***P***	**OR (95% CI)**	***P***	**OR (95% CI)**	***P***
**From age 25 years to 10 years before survey**							
Stable Normal (ref)	778/9022	1.00		1.00		1.00	
Maximum Overweight	743/9483	0.95 (0.82, 1.09)	0.451	0.97 (0.84, 1.13)	0.739	1.09 (0.93, 1.27)	0.278
Non-obesity to Obesity	601/5300	1.40 (1.20, 1.62)	<0.001	1.32 (1.13, 1.54)	0.001	1.49 (1.27, 1.75)	<0.001
Obesity to Non-obesity	35/268	1.72 (1.04, 2.84)	0.035	1.98 (1.20, 3.28)	0.008	1.71 (0.99, 2.95)	0.054
Stable Obesity	191/1670	1.17 (0.94, 1.47)	0.158	1.37 (1.09, 1.71)	0.007	1.45 (1.15, 1.83)	0.002
**From 10 years before survey to survey**							
Stable Normal (ref)	425/5236	1.00		1.00		1.00	
Maximum Overweight	625/8851	0.94 (0.79, 1.11)	0.459	0.98 (0.83, 1.17)	0.847	1.07 (0.91, 1.27)	0.412
Non-obesity to Obesity	506/4686	1.56 (1.30, 1.87)	<0.001	1.59 (1.32, 1.91)	<0.001	1.76 (1.47, 2.11)	<0.001
Obesity to Non-obesity	147/1317	1.55 (1.15, 2.08)	0.004	1.55 (1.15, 2.09)	0.004	1.52 (1.11, 2.08)	0.009
Stable Obesity	645/5653	1.49 (1.25, 1.77)	<0.001	1.49 (1.25, 1.77)	<0.001	1.75 (1.47, 2.08)	<0.001
**From age 25 years to survey**							
Stable Normal (ref)	494/5860	1.00		1.00		1.00	
Maximum Overweight	664/9187	0.94 (0.79, 1.13)	0.520	1.00 (0.84, 1.20)	0.982	1.12 (0.94, 1.34)	0.207
Non-obesity to Obesity	964/8758	1.49 (1.28, 1.74)	<0.001	1.48 (1.26, 1.73)	<0.001	1.73 (1.48, 2.02)	<0.001
Obesity to Non-obesity	39/357	1.13 (0.69, 1.84)	0.625	1.33 (0.82, 2.17)	0.243	1.14 (0.68, 1.91)	0.622
Stable Obesity	187/1581	1.38 (1.10, 1.73)	0.006	1.64 (1.31, 2.05)	<0.001	1.84 (1.46, 2.31)	<0.001

^a^All estimates accounted for complex survey designs.

^b^Stable Normal pattern (<25.0 at both times), Maximum Overweight pattern (25.0–29.9 at either time but not ≥30.0 at the other time), Non-obesity to Obesity pattern (<30.0 at younger age and ≥30.0 later), Obesity to Non-obesity pattern (≥30.0 at younger age and <30.0 later), and Stable Obesity (≥30.0 at both times).

^c^Model 1 was unadjusted.

^d^Model 2 was adjusted for age and sex.

^e^Model 3 was additionally adjusted for race/ethnicity, education level, family income-poverty ratio level, smoking status, and drinking status.

**Fig. 1:**
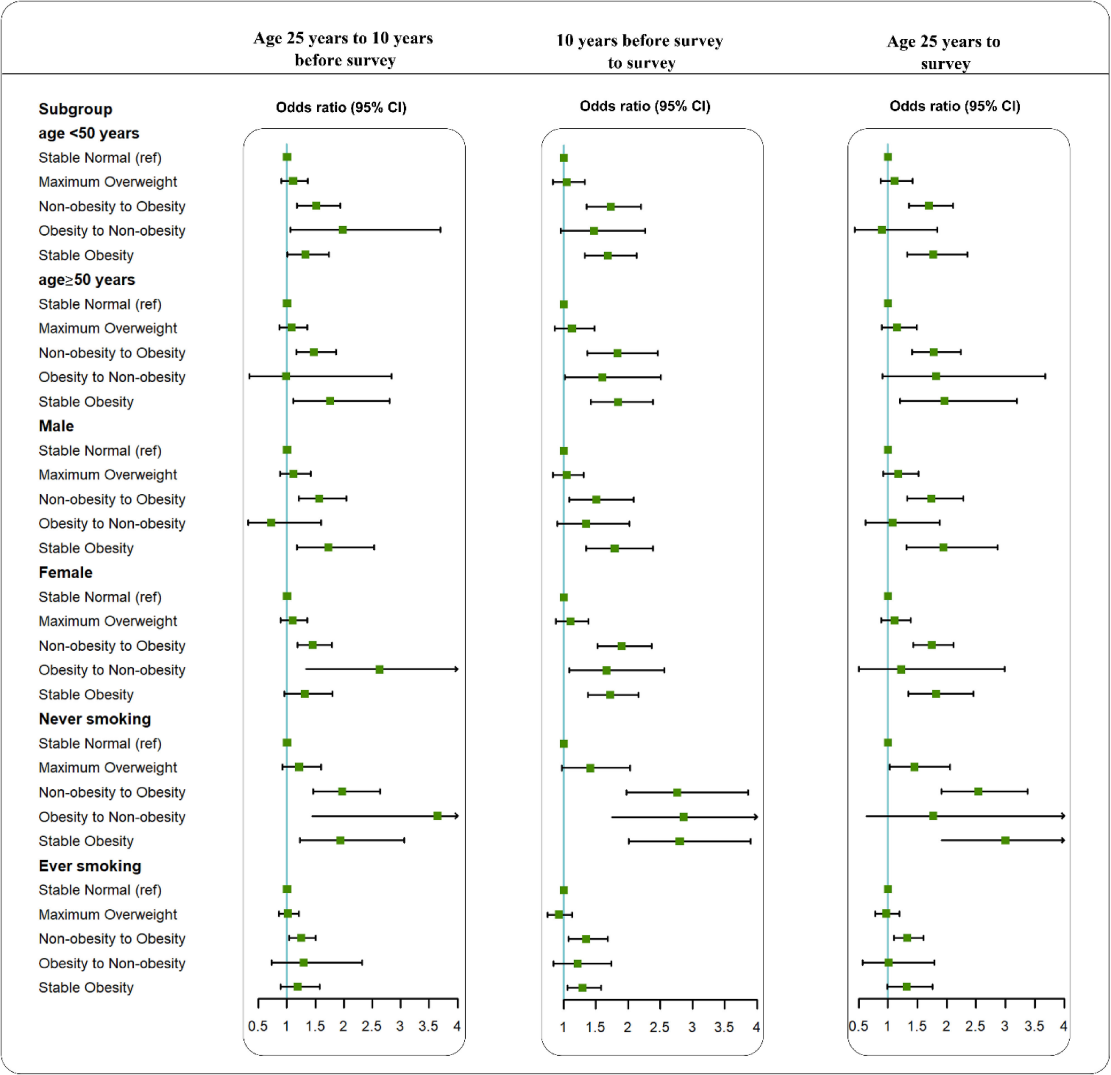
Associations between weight change patterns across adulthood and risk of COPD in NHANES 1999–2018. All estimates accounted for complex survey design of NHANES. Risk estimates were adjusted for age (not adjusted in subgroup analysis by age), sex (not adjusted in subgroup analysis by sex), race/ethnicity, education level, family income-poverty ratio level, smoking status (not adjusted in subgroup analysis by smoking status), and drinking status.

In the stratified analyses, we found significant interactions with smoking status (Fig. 1). In the analysis of weight change from age 25 years to 10 years before the survey, the effects of weight gain on COPD were stronger among never-smokers (OR = 1.97, 95% CI = 1.47 to 2.64) compared with ever-smokers (OR = 1.25, 95% CI = 1.04 to 1.51), and *p* for interaction was 0.032. We also found similar results according to the weight change pattern in the other two time periods.

### The relationship between absolute weight change and COPD

**Fig. 2:**
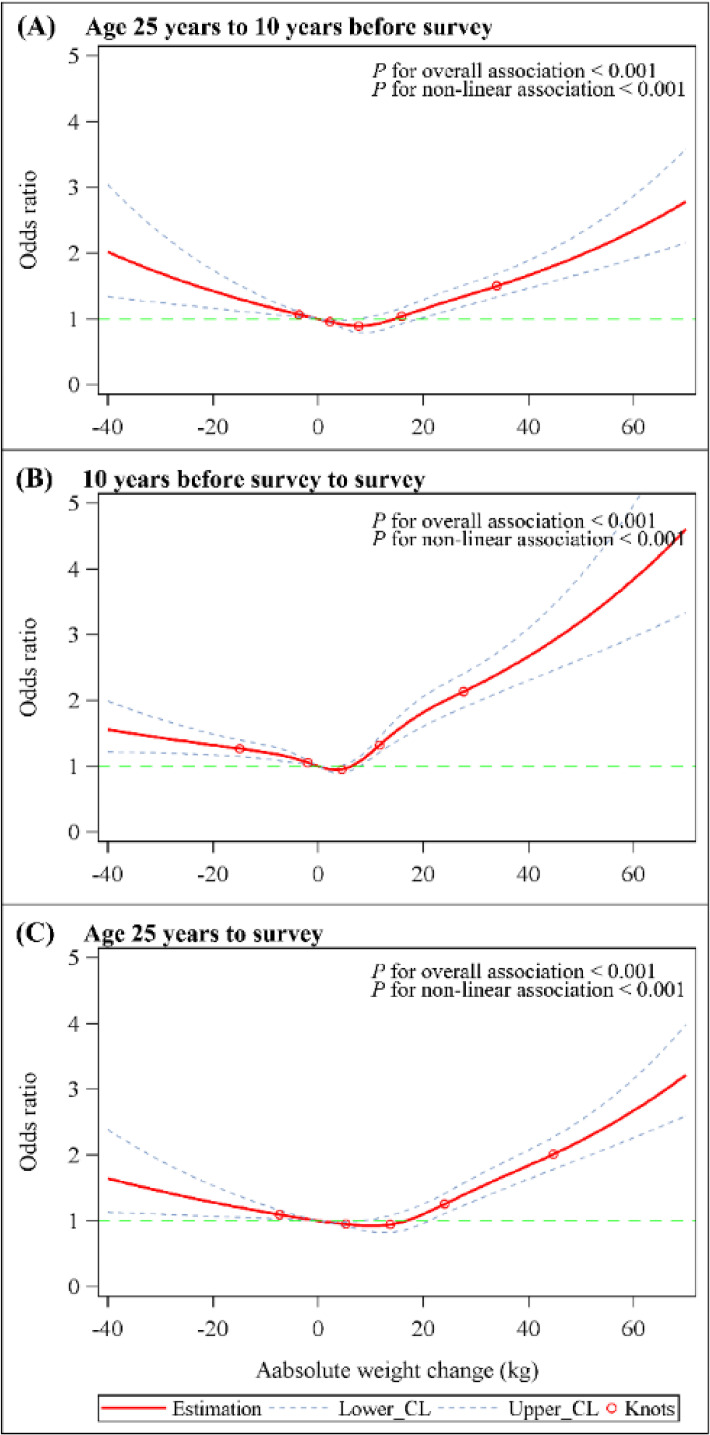
Dose-response association between absolute weight change across adulthood and risk of COPD. Associations were examined by multivariable Logistic regression models based on restricted cubic splines. Red solid line represents estimates of odds ratios (reference value for absolute weight change: 0 kg). Blue dashed line represents 95% confidence intervals. Red circle represents knots located at the 5th, 25th, 50th, 75th and 95th percentiles of the distribution of absolute weight change. Risk estimates were adjusted for age, sex, race/ethnicity, education level, family income-poverty ratio level, smoking status, and drinking status. For weight change from age 25 years to 10 years before survey or to survey, BMI at age 25 was also adjusted for. For weight change from 10 years before survey to survey, BMI at 10 years previously was also adjusted for. *P* values for overall association and *P* values for non-linear association were all <0.001 in three periods.

By evaluating the absolute weight change over three periods, we found a U-shaped association between the risk of COPD occurrence and absolute weight change in adulthood (Fig. 2). Among the five groups of absolute weight changes, the extreme weight gain group (weight gain ≥ 20 kg) showed a more significant correlation with COPD, with an OR of 1.43 (95% CI = 1.21 to 1.70) from age of 25 years to 10 years before the survey, 2.52 (95% CI = 1.99 to 3.18) in the 10 years period before the survey, and 1.97 (95% CI = 1.55 to 2.51) from age of 25 years to the survey (Table 3). Moderate to large weight gain (10 kg ≤ weight gain < 20.0 kg) also showed the same correlation. Compared with stable weight group (weight change within 2.5 kg), mild to moderate weight gain (2.5 kg ≤ weight gain < 10.0 kg) showed no correlation with COPD in the three time periods. For the weight loss group (weight loss ≥ 2.5 kg), the OR for COPD was 1.56 (95% CI = 1.15 to 2.12) from age of 25 years to the survey, 1.24 (95% CI = 0.99 to 1.56) from age of 25 years to 10 years before the survey, and 1.18 (95% CI = 0.98 to 1.43) in the 10 years period before the survey. When we restricted the analysis to participants who had a BMI of <25 at 25 years of age or at 10 years before survey, the results were still similar, indicating that our estimation was not driven by participants with high weight levels of weight gain (Fig. S3). When stratified by age, gender and smoking status, these associations were similar to our main results (Fig. S3).

**Table 3:** Odd ratios (OR) and 95% confidence intervals (CIs) of COPD with absolute weight change groups.^a^

**Weight Change Patterns^b^**	**No. of COPD/No. ** **of participants**	**Model 1^c^**		**Model 2^d^**		**Model 3^e^**	

		**OR (95% CI)**	***P***	**OR (95% CI)**	***P***	**OR (95% CI)**	***P***
**From age 25 years to 10 years before survey**							
Weight loss ≥2.5 kg	181/1424	1.54 (1.24, 1.92)	<0.001	1.45 (1.17, 1.81)	0.001	1.24 (0.99, 1.56)	0.063
Weight change within 2.5 kg (ref)	499/5940	1.00		1.00		1.00	
Weight gain ≥2.5 kg and <10 kg	586/8083	0.82 (0.69, 0.96)	0.012	0.76 (0.65, 0.89)	0.001	0.88 (0.74, 1.03)	0.117
Weight gain ≥10 kg and <20 kg	505/5861	1.07 (0.90, 1.26)	0.466	0.90 (0.75, 1.08)	0.255	1.06 (0.89, 1.27)	0.532
Weight gain ≥20 kg	577/4435	1.60 (1.37, 1.88)	<0.001	1.27 (1.08, 1.51)	0.005	1.43 (1.21, 1.70)	<0.001
**From 10 years before survey to survey**							
Weight loss ≥2.5 kg	636/5966	1.62 (1.35, 1.93)	<0.001	1.62 (1.36, 1.94)	<0.001	1.18 (0.98, 1.43)	0.081
Weight change within 2.5 kg (ref)	307/4700	1.00		1.00		1.00	
Weight gain ≥2.5 kg and <10 kg	517/7447	0.98 (0.82, 1.16)	0.785	1.00 (0.84, 1.20)	0.962	1.00 (0.84, 1.20)	0.975
Weight gain ≥10 kg and <20 kg	467/4846	1.47 (1.21, 1.77)	<0.001	1.57 (1.29, 1.91)	<0.001	1.46 (1.20, 1.79)	<0.001
Weight gain ≥20 kg	421/2784	2.65 (2.16, 3.24)	<0.001	2.89 (2.32, 3.60)	<0.001	2.52 (1.99, 3.18)	<0.001
**From age 25 years to survey**							
Weight loss ≥2.5 kg	303/2497	2.06 (1.53, 2.77)	<0.001	2.10 (1.56, 2.82)	<0.001	1.56 (1.15, 2.12)	0.004
Weight change within 2.5 kg (ref)	155/2201	1.00		1.00		1.00	
Weight gain ≥2.5 kg and <10 kg	351/5316	1.03 (0.80, 1.33)	0.800	1.04 (0.81, 1.34)	0.734	1.14 (0.88, 1.47)	0.316
Weight gain ≥10 kg and <20 kg	532/7134	1.20 (0.95, 1.52)	0.121	1.16 (0.92, 1.47)	0.208	1.28 (1.01, 1.62)	0.045
Weight gain ≥20 kg	1007/8595	1.99 (1.58, 2.51)	<0.001	1.81 (1.43, 2.29)	<0.001	1.97 (1.55, 2.51)	<0.001

^a^All estimates accounted for complex survey designs.

^b^Absolute weight change: weight loss of at least 2.5 kg, weight change within 2.5 kg, weight gain of at least 2.5 kg but less than 10.0 kg, weight gain of at least 10 kg but less than 20.0 kg, and weight gain of at least 20.0 kg.

^c^Model 1 was unadjusted.

^d^Model 2 was adjusted for age and sex.

^e^Model 3 was additionally adjusted for race/ethnicity, education level, family income-poverty ratio level, smoking status, and drinking status.

## Discussion

In this large, nationally representative retrospective study of adults in the United States, we found that both stable obesity and weight gain in adulthood were associated with an increased risk of COPD incidence. Extreme weight gain of more than 20 kg in adulthood was associated with a higher risk of COPD compared to those with stable weight. The impact of weight change patterns on the risk of COPD was more pronounced in non-smoker than former smoker. In addition, there was a significant nonlinear dose-response relationship between absolute weight gain and COPD incidence over the three time intervals. Our findings emphasize the importance of maintaining stable weight in adulthood, particularly in preventing stable obesity or weight gain in adulthood, which is closely related to reducing the risk of COPD later in life.

It is well-established that a high BMI in midlife affects nearly all physiological functions and increases the risk of developing multiple disease conditions, including COPD and other cardiovascular disease (CVD) [22]. The relationship between weight change and COPD still deserves more research attention. Zhang et al. analyzed the BMI of more than 1.5 million participants and found that BMI was associated with the risk of COPD, underweight might increase the risk of COPD, overweight and obesity might reduce the risk of COPD [23]. The results of this systematic review and meta-analysis were inconsistent with our findings. On the one hand, our study is based on a retrospective study of the adult population in the United States, while Zhang et al. surveyed over 1.5 million participants from different regions and ethnic groups, which may introduce more heterogeneity. On the other hand, unlike its cross-sectional research method, our study used a ‘longitudinal design’, which may better capture the temporal relationship between weight changes and COPD development. In addition, the prevalence and risk factors of COPD may vary by country and time due to differences in the “time period” and “geographical scope” of data sources. In future, more researches are needed on the causes of weight change to explain the association between weight change and COPD risk to validate our findings. On the other hand, our results did not find a significant association between weight loss and the risk of COPD, which might be due to the relatively small proportion of people transitioning from obesity to non-obesity (with this population accounting for 0.9% from the age of 25 years to the 10 years before the survey, 4.34% from 10 years before survey to survey, and 1.06% from the age of 25 years to the survey). Therefore, this result should be interpreted with caution.

In addition, there was also a correlation between obesity and changes in lung function. Obesity may contribute to both obstructive (COPD) and restrictive lung diseases through several mechanisms. Obesity can lead to systemic inflammatory responses, increase oxidative stress, and promote the release of adipokines. These factors may exacerbate pulmonary inflammation and airway remodeling, thereby contributing to the development of COPD. A national 7-year follow-up study in the UK found that changes in weight or body mass were related to changes in FEV1 [24]. A health survey of 650,000 Canadian adults found that the probability of COPD patients coexisting with obesity was 24.6%, while the probability of non-COPD patients coexisting with obesity was 17.1% [25]. Costa Melo et al. systematically analyzed the relationship between obesity and lung function and found that compared with normal weight people, the total lung capacity, forced lung capacity and forced expiratory volume in the first second of obese people were reduced, suggesting that obesity might play a promoting role in the occurrence of COPD, and further demonstrating the correlation between body weight pattern and COPD [26]. Our findings indicated a U-shaped relationship between absolute weight change in the survey and the risk of COPD. The relationship between obesity and COPD might vary depending on educational background, ethnicity, and geography. Overweight and obesity were more common in middle-aged and elderly COPD patients. A randomized controlled trial of low-intensity lifestyle intervention in overweight or obese COPD patients found that moderate weight loss did not lead to significant clinical improvement in physical function and dyspnea [27]. These evidences suggested that weight loss after COPD had little impact on respiratory health. We support that maintaining a healthy and stable body weight in adulthood and avoiding obesity are important strategies to reduce future COPD risk.

The mechanisms by which obesity causes the onset of COPD is still unclear, and several possible mechanisms had been suggested to explain the relationship between obesity and COPD. For example, obese patients consume more oxygen and have an increased demand for lung support, while adipose tissue accumulation impairs individual ventilation function [28]. On the other hand, obese patients have limited thoracic motion, leading to impaired diaphragmatic excursion and reduced thoracic compliance, especially in severely obese patients [29, 30]. Some studies had also found that elevated levels of various inflammatory markers in obese patients were capable of triggering local or systemic inflammatory responses, which also played promoting roles in the onset of COPD [31]. In the following research, we also need to further investigate how obesity damages lung function through various mechanisms. Furthermore, it may be more meaningful to consider physical activity as an additional covariate.

Smoking is the most widely recognized risk factor for COPD, but 23% of COPD patients still never smoke [32]. Compared to smokers, our findings also found that weight change had a more pronounced effect on never-smokers. This may be due to the fact that other risk factors such as weight changes play a more significant and measurable role in the development of COPD when smoking is not a confounding factor. In addition, never-smokers may be less likely to be screened for COPD unless symptomatic, and when they are diagnosed, obesity-related symptoms (like dyspnea) may already be more severe or have progressed. This may create an apparent stronger association between weight change and COPD prevalence in this group [32, 33]. Fuller-Thomson et al. found that, in a study targeting a non-Hispanic white population of lifelong non-smokers, the prevalence of COPD had increased from 2.5% in male with a healthy body weight (BMI < 25 kg/m^2^) and 3.5% in female to 7.6% in male with a BMI ≥ 40 kg/m^2^ and 13.4% in female [33]. A large cohort study conducted by Gundula et al. found that obesity, particularly abdominal obesity, was associated with an increased risk of COPD and that there was a significant positive correlation between BMI and COPD in subjects who had never smoked [34]. This study provided important support for the increased risk of COPD in individuals with obesity. Our results were consistent with these studies and suggested a greater need for COPD risk awareness, regular monitoring of changes in lung function, and screening for COPD in patients with stable obesity or weight gain who have never smoked.

### Strengths and limitations

One of the strengths in our study is that we conducted a nationally representative large-scale retrospective study to investigate the association between adult weight change and the risk of COPD. Additionally, in our study, absolute weight changes were also considered as continuous variables to analyze the relationship between the absolute weight change group and COPD risk. We also used restricted cubic spline with 5 nodes to evaluate the dose-response relationship. Our study identified weight change as a risk factor for COPD through five patterns of weight change, and this finding would help alleviate the disease burden of chronic respiratory diseases in the future. However, there are a few limitations to be considered. Firstly, we used self-reported weight data from NHANES at age of 25 years and the 10 years before the survey, which might have introduced misclassification bias. Nevertheless, self-reported early BMI and weight recall had been shown to be valid measures for assessment [35]. Secondly, due to the lack of data at different time points, we did not assess the association of other obesity-related measures such as changes in waist circumference and fat mass with COPD. Examining duplicate data on these biomarkers may provide a more comprehensive explanation for obesity and COPD risk. It is worth noting that the United States is one of the countries with the highest average obesity rates globally, which may lead to differences in our results compared to studies conducted in populations with lower BMI distributions. We need to explore whether similar patterns of weight changes will have similar health effects on other populations, particularly in countries with lower obesity rates.

## Conclusions

Our study suggested that stable obesity and weight gain in adulthood were associated with an increased risk of COPD compared to those who maintain a normal weight, and that the association between weight gain and the incidence of COPD appears closer in patients who have never smoked.

## Abbreviations

COPD: chronic obstructive pulmonary disease
NHANES: National Health and Nutrition Examination Survey
BMI: body mass index
CRD: chronic respiratory disease
NCHS: National Center for Health Statistics
PIR: family income-to poverty ratio
PSUs: primary sampling units
SE: standard error

## Supplementary information

**Additional file 1:** **Figure S1.** A flow chart of inclusion and exclusion of study participants. **Figure S2.** Dose-response association between BMI and risk of COPD. **Figure S3.** Associations between absolute weight change groups across adulthood and risk of COPD in NHANES 1999–2018. **Table S1.** According to weight change patterns from 10 years before survey to survey. **Table S2.** According to weight change patterns from age 25 years to survey. **Table S3.** Odd ratios (OR) and 95% confidence intervals (CIs) of COPD with BMI status.

## Declarations

### Availability of data and materials

The datasets generated and analyzed in the current study are available at NHANES website: https://www.cdc.gov/nchs/nhanes/index.htm.

## References

[1] Ferrera MC, Labaki WW, Han MK. Advances in Chronic Obstructive Pulmonary Disease. Annu Rev Med. 2021;72:119–34. 33502902 10.1146/annurev-med-080919-112707 PMC8011854

[2] Global burden of chronic respiratory diseases and risk factors, 1990–2019: an update from the Global Burden of Disease Study 2019. EClinicalMedicine. 2023;59:101936. 37229504 10.1016/j.eclinm.2023.101936 PMC7614570

[3] Katz P, Iribarren C, Sanchez G, Blanc PD. Obesity and Functioning Among Individuals with Chronic Obstructive Pulmonary Disease (COPD). COPD. 2016;13(3):352–9. 26683222 10.3109/15412555.2015.1087991 PMC4951092

[4] McDonald VM, Wood LG, Holland AE, Gibson PG. Obesity in COPD: to treat or not to treat? Expert Rev Respir Med. 2017;11(2):81–3. 27910701 10.1080/17476348.2017.1267570

[5] Lambert AA, Putcha N, Drummond MB, Boriek AM, Hanania NA, Kim V, Kinney GL, McDonald MN, Brigham EP, Wise RA, . Obesity Is Associated With Increased Morbidity in Moderate to Severe COPD. Chest. 2017;151(1):68–77. 27568229 10.1016/j.chest.2016.08.1432 PMC5310126

[6] Holtjer JCS, Bloemsma LD, Beijers RJHCG, Cornelissen MEB, Hilvering B, Houweling L, Vermeulen RCH, Downward GS, Maitland-Van der Zee AH; P4O2 consortium. Identifying risk factors for COPD and adult-onset asthma: an umbrella review. Eur Respir Rev. 2023 May 3;32(168):230009. 37137510 10.1183/16000617.0009-2023 PMC10155046

[7] Jung JW, Yoon SW, Lee GE, Shin HG, Kim H, Shin JW, Park IW, Choi BW, Kim JY. Poor nutritional intake is a dominant factor for weight loss in chronic obstructive pulmonary disease. Int J Tuberc Lung Dis. 2019;23(5):631–7. 31097074 10.5588/ijtld.18.0456

[8] Kwan HY, Maddocks M, Nolan CM, Jones SE, Patel S, Barker RE, Kon SSC, Polkey MI, Cullinan P, Man WD. The prognostic significance of weight loss in chronic obstructive pulmonary disease-related cachexia: a prospective cohort study. J Cachexia Sarcopenia Muscle. 2019;10(6):1330–8. 31207189 10.1002/jcsm.12463 PMC6903442

[9] Hallin R, Koivisto-Hursti UK, Lindberg E, Janson C. Nutritional status, dietary energy intake and the risk of exacerbations in patients with chronic obstructive pulmonary disease (COPD). Respir Med. 2006;100(3):561–7. 16019198 10.1016/j.rmed.2005.05.020

[10] Agustí AG, Noguera A, Sauleda J, Sala E, Pons J, Busquets X. Systemic effects of chronic obstructive pulmonary disease. Eur Respir J. 2003;21(2):347–60. 12608452 10.1183/09031936.03.00405703

[11] Vandenbergh E, Van de Woestijne KP, Gyselen A. Weight changes in the terminal stages of chronic obstructive pulmonary disease. Relation to respiratory function and prognosis. Am Rev Respir Dis. 1967;95(4):556–66. 6021915 10.1164/arrd.1967.95.4.556

[12] Wilson DO, Rogers RM, Wright EC, Anthonisen NR. Body weight in chronic obstructive pulmonary disease. The National Institutes of Health Intermittent Positive-Pressure Breathing Trial. Am Rev Respir Dis. 1989;139(6):1435–8. 2658702 10.1164/ajrccm/139.6.1435

[13] Gray-Donald K, Gibbons L, Shapiro SH, Macklem PT, Martin JG. Nutritional status and mortality in chronic obstructive pulmonary disease. Am J Respir Crit Care Med. 1996;153(3):961–6. 8630580 10.1164/ajrccm.153.3.8630580

[14] Schols AM, Slangen J, Volovics L, Wouters EF. Weight loss is a reversible factor in the prognosis of chronic obstructive pulmonary disease. Am J Respir Crit Care Med. 1998;157(6 Pt 1):1791–7. 9620907 10.1164/ajrccm.157.6.9705017

[15] Zipf G, Chiappa M, Porter KS, Ostchega Y, Lewis BG, Dostal J. National health and nutrition examination survey: plan and operations, 1999–2010. Vital Health Stat 1. 2013;(56):1–37. 25078429

[16] Stokes A, Collins JM, Grant BF, Scamuffa RF, Hsiao CW, Johnston SS, Ammann EM, Manson JE, Preston SH. Obesity Progression Between Young Adulthood and Midlife and Incident Diabetes: A Retrospective Cohort Study of U.S. Adults. Diabetes Care. 2018;41(5):1025–31. 29506982 10.2337/dc17-2336 PMC5911788

[17] Chen C, Ye Y, Zhang Y, Pan XF, Pan A. Weight change across adulthood in relation to all cause and cause specific mortality: prospective cohort study. BMJ. 2019;367:l5584. 31619383 10.1136/bmj.l5584 PMC6812615

[18] Wang T, Zhou Y, Kong N, Zhang J, Cheng G, Zheng Y. Weight gain from early to middle adulthood increases the risk of incident asthma later in life in the United States: a retrospective cohort study. Respir Res. 2021;22(1):139. 33952267 10.1186/s12931-021-01735-7 PMC8097961

[19] Wang L, Yi J, Guo J, Ren X. Weigh change across adulthood is related to the presence of NAFLD: results from NHANES III. J Transl Med. 2023 Feb 23;21(1):142. 36823668 10.1186/s12967-023-04007-8 PMC9951528

[20] Zhao F, Zhao Q, Wang H, Wang K, Kong S, Ma P, Wang X. Weight changes from early to middle adulthood and cardiometabolic multimorbidity later in life among middle-aged and older adults: a retrospective cohort study from the NHANES 1999–2018. Front Endocrinol (Lausanne). 2024 Feb 19;15:1306551. 38440787 10.3389/fendo.2024.1306551 PMC10910024

[21] Wang T, Dai B, Shi H, Li H, Fan K, Zhang D, Zhou Y. Weight change across adulthood in relation to the risk of depression. Front Psychol. 2023 Aug 9;14:1108093. 37621933 10.3389/fpsyg.2023.1108093 PMC10446764

[22] Ojalehto E, Zhan Y, Jylhävä J, Reynolds CA, Dahl Aslan AK, Karlsson IK. Genetically and environmentally predicted obesity in relation to cardiovascular disease: a nationwide cohort study. EClinicalMedicine. 2023 Apr 6;58:101943. 37181410 10.1016/j.eclinm.2023.101943 PMC10166783

[23] Zhang X, Chen H, Gu K, Chen J, Jiang X. Association of Body Mass Index with Risk of Chronic Obstructive Pulmonary Disease: A Systematic Review and Meta-Analysis. COPD. 2021;18(1):101–13. 33590791 10.1080/15412555.2021.1884213

[24] Carey IM, Cook DG, Strachan DP. The effects of adiposity and weight change on forced expiratory volume decline in a longitudinal study of adults. Int J Obes Relat Metab Disord. 1999;23(9):979–85. 10490805 10.1038/sj.ijo.0801029

[25] Vozoris NT, O’Donnell DE. Prevalence, risk factors, activity limitation and health care utilization of an obese, population-based sample with chronic obstructive pulmonary disease. Can Respir J. 2012;19(3):e18–24. 22679617 10.1155/2012/732618 PMC3418099

[26] Melo LC, Silva MA, Calles AC. Obesity and lung function: a systematic review. Einstein (Sao Paulo). 2014;12(1):120–5. 24728258 10.1590/S1679-45082014RW2691 PMC4898251

[27] Au DH, Gleason E, Hunter-Merrill R, Barón AE, Collins M, Ronneberg C, Lv N, Rise P, Wai TH, Plumley R, . Lifestyle Intervention and Excess Weight in Chronic Obstructive Pulmonary Disease (COPD): INSIGHT COPD Randomized Clinical Trial. Ann Am Thorac Soc. 2023;20(12):1743–51. 37769182 10.1513/AnnalsATS.202305-458OC PMC10704228

[28] Poulain M, Doucet M, Major GC, Drapeau V, Sériès F, Boulet LP, Tremblay A, Maltais F. The effect of obesity on chronic respiratory diseases: pathophysiology and therapeutic strategies. CMAJ. 2006;174(9):1293–9. 16636330 10.1503/cmaj.051299 PMC1435949

[29] Ray CS, Sue DY, Bray G, Hansen JE, Wasserman K. Effects of obesity on respiratory function. Am Rev Respir Dis. 1983;128(3):501–6. 6614644 10.1164/arrd.1983.128.3.501

[30] Biring MS, Lewis MI, Liu JT, Mohsenifar Z. Pulmonary physiologic changes of morbid obesity. Am J Med Sci. 1999;318(5):293–7. 10555090 10.1097/00000441-199911000-00002

[31] Ferrante AW Jr. Obesity-induced inflammation: a metabolic dialogue in the language of inflammation. J Intern Med. 2007;262(4):408–14. 17875176 10.1111/j.1365-2796.2007.01852.x

[32] Celli BR, Halbert RJ, Nordyke RJ, Schau B. Airway obstruction in never smokers: results from the Third National Health and Nutrition Examination Survey. Am J Med. 2005;118(12):1364–72. 16378780 10.1016/j.amjmed.2005.06.041

[33] Fuller-Thomson E, Howden KEN, Fuller-Thomson LR, Agbeyaka S. A Strong Graded Relationship between Level of Obesity and COPD: Findings from a National Population-Based Study of Lifelong Nonsmokers. J Obes. 2018;2018:6149263. 30584475 10.1155/2018/6149263 PMC6280223

[34] Behrens G, Matthews CE, Moore SC, Hollenbeck AR, Leitzmann MF. Body size and physical activity in relation to incidence of chronic obstructive pulmonary disease. CMAJ. 2014;186(12):E457–69. 25002559 10.1503/cmaj.140025 PMC4150732

[35] De Rubeis V, Bayat S, Griffith LE, Smith BT, Anderson LN. Validity of self-reported recall of anthropometric measures in early life: A systematic review and meta-analysis. Obes Rev. 2019;20(10):1426–40. 31184422 10.1111/obr.12881

